# Social Responses to Epidemics Depicted by Cinema 

**DOI:** 10.3201/eid2602.181022

**Published:** 2020-02

**Authors:** Qijun Han, Daniel R. Curtis

**Affiliations:** Nanjing University of Science and Technology, Nanjing, China (Q. Han);; Erasmus University Rotterdam, Rotterdam, the Netherlands (D.R. Curtis)

**Keywords:** Cinema, epidemics, morality, disorder, resistance, films

## Abstract

Films illustrate 2 ways that epidemics can affect societies: fear leading to a breakdown in sociability and fear stimulating preservation of tightly held social norms. The first response is often informed by concern over perceived moral failings within society, the second response by the application of arbitrary or excessive controls from outside the community.

Films related to themes of disease, infection, and contagion often fall into 1 of 3 broad categories connected to fantasy, science fiction, or horror: apocalyptic destruction or near destruction of the whole of humanity, rising concerns over bioterrorism, and the rise of an undead or form of zombie existence (*1*). Although films traditionally deal sensitively or realistically with the topic of HIV/AIDS (e.g., *Dallas Buyers Club*,* Philadelphia*,* And the Band Played On*,* Kids*), often through melodrama, fewer films have dealt with other epidemic diseases, as either direct subject material or background context. Of these more realistic or semirealistic films about epidemics, scholarly literature has focused on the inadequacy of capturing the correct science behind disease transmission, spread, and illness (*2*–*4*) or anachronistic characters in films concerning historical epidemics (*5*). Notwithstanding broader discussions of society–disease interaction in the media (*6*), the social responses to various diseases portrayed in films have been discussed less frequently.

In this article, we bring together a sample of films that we manually selected from a consolidated database of epidemic-related films built from assorted scholarly literature and catalogs (*2*,*4*,*5*,*7*–*9*). This database was supplemented by accessing “Films about viral outbreaks” on Wikipedia (https://en.wikipedia.org/wiki/Category:Films_about_viral_outbreaks) and a list of “Apocalyptic, epidemic, pandemic and disaster movies” on the Internet Movie Database (https://www.imdb.com/list/ls058975821). We manually selected the films and eliminated those that corresponded to the 3 broad categories mentioned above (i.e., those that are overly fantastical, not based or loosely based on an actual epidemic disease) and focused on films that pay explicit attention to social responses to disease (rather than being a peripheral backdrop to an unrelated story). Furthermore, the absence of comprehensive indexing of films containing narratives around epidemics makes construction of a systematic sample impractical (*4*).

Scholarly literature that focuses on contemporary disease psychology holds central a connection between fear, panic, and epidemics (*10*), often focusing on the unique characteristics of infectious diseases themselves (*11*). Indeed, in an article about the psychosocial effects of diseases, the disproportionate degree of fear was describe as connected to the fact that “it is transmitted rapidly and invisibly; historically, it has accounted for major morbidity and mortality; old forms re-emerge and new forms emerge; and both the media and society are often in awe” (*12*). However, after analyzing this selection of epidemics-related films, we suggest that although social reactions such as panic (an emotive response caused by fear) are typically found in films concerning epidemics, films also remind us that the fear seen during epidemics is often little associated with the disease itself. In fact, films show that epidemics can push societies in 2 directions: fear leading to a breakdown in sociability, but also fear stimulating the preservation of tightly held social norms. The first social response to epidemics is often informed by concern over broader moral failings within society at large, leading, for example, to violence or scapegoating. In accordance with the “outbreak narrative,” a concept developed by Priscilla Wald, a fear of the spread of disease is developed in only 1 direction, from marginalized, deviant, or underdeveloped groups to native, mainstream, or developed society (*6*). In recent films, this kind of orientalization (perpetuating stereotypes about Middle Eastern, Asian, and North African societies) and othering (viewing or treating others as intrinsically different from and alien to oneself) has been taken a step further as traditional or underdeveloped societies are heroically saved by outsiders. The second social response to epidemics is often informed by the perceived application of arbitrary or excessive controls from above or outside the community in question. Films have shown that epidemics produce active responses such as resistance or unrest—sometimes violent—to paradoxically retain aspects of normal life under threat (often from elites and authorities), such as perceived freedoms and liberties and customary traditions and practices.

## Social Morality during Epidemics in Cinema

Many films dealing with epidemics have tended to see panic as an inevitable social response; their main focus has been the process of authorities withholding information to guard against chaos or the circulation of misinformation by the media. For example, in *Panic on the Streets* (1950, directed by Elia Kazan), to avoid mass panic across the city of New Orleans, the US Public Health Service and the police agree to not notify the press of a death resulting from pneumonic plague. Appearing in the same year, *The Killer That Stalked New York* (1950, Earl McEvoy) was based on an actual threat of smallpox that occurred in New York in 1947. In the film, public health officials develop a widespread vaccination program, but after the necessary serum runs out, the city descends into mass panic—after the authorities tried to cover up this information. In the UK film *80,000 Suspects* (1963, Val Guest), the doctor tackling an outbreak of smallpox uses a quarantining process with the explicitly mentioned goal of reducing the chances of public panic. In *Morte a Venezia* (1971, Luchino Visconti), a film based on the 1912 novel by German author Thomas Mann, *Der Tod in Venedig* [*Death in Venice*], the city authorities do not inform those on vacation of cholera problems within the city for fear they will frantically leave—an approach also taken by town officials in John Ford’s depiction of a community during a typhoid epidemic, *Dr. Bull* (1933). Another common feature is defiance of the film’s protagonists against a perceived lack of official information. For example, in *Quiet Killer *(1992, Sheldon Larry), when the doctor realizes that her patient has succumbed to plague, she tries to push authorities to warn the citizens of New York, against considerable reluctance from the mayor, who envisages widespread panic.

Other films, however, have gone further and tried to examine some of the causes of this fear and panic; in many films, the roots lie in society’s response to perceived declines in social morality. One of the earliest examples is *Die Pest in Florenz* [*The Plague of Florence*] (1918, Fritz Lang), which focused on the real outbreak of the Black Death in Florence in the mid-14th century and portrayed death from plague as a response to immoral behavior and sexual debauchery. The connection between disease and deteriorating social morality came from actual observations of contemporaries at the time, for example, the views of Giovanni Villani (*Nuova Cronica*) and Giovanni Boccaccio (*Decameron*). In several films, the plague became used as an explicit punishment for immorality and wrongdoing: *The Pied Piper* (1972, Jacques Demy), *The Hour of the Pig* (1993, Leslie Megahey), and especially *Anazapta *(2002, Alberto Sciamma), in which plague was a supposed consequence of the brutal rape of a lord’s wife by the village. 

Similar kinds of existentialist angst and pessimism over social values in connection with the Black Death were later famously exploited by Ingmar Bergman in *The Seventh Seal* (1957), Lars von Trier in *Epidemic* (1987), Luis Puenzo in *The Plague* (1992), and Christopher Smith in *The Black Death* (2010). Those 4 films focus especially on intolerance in the form of scapegoating and persecution of women as witches, but visualization of dread had already appeared earlier in lesser known films, such as *Singoalla* (1949, Christian-Jaque), *Häxan* (1922, Benjamin Christensen), and *Skeleton on Horseback* (1937, Hugo Haas). In the film *Trollsyn *(1994, Ola Solum), set in Norway, the initial stage of the Black Death outbreak is presented as mass hysteria among the villagers: crying and shouting, desperate prayers, suicides, and frantic searching for buboes and panicked movements. Also, in reference to social decline, one scene shows simultaneously the aggressive inquisition of a female scapegoat while amid the chaos a couple are having sex and others have taken off their clothes and are rolling down a grassy hill.

Many films focusing on outbreaks of epidemic disease focus on outbreaks of senseless violence indicative of a society completely out of control. In *The Horseman on the Roof* (1995, Jean-Paul Rappeneau), set during a cholera epidemic in 19th-century Manosque, southern France, the film’s protagonist, Angelo, is captured by a paranoid mob who accuse him of poisoning the town fountain and take him to the authorities. Elsewhere, in *the Masque of the Red Death* (1964, Roger Corman [adapted in 1989 by Larry Brand/Jeffrey Delman]), which focuses on a fictional disease with loose parallels to plague, rural villagers become increasingly desperate and seek to escape the devastating death, only to have soldiers shoot them down by crossbow. In *Jezebel* (1939, William Wyler), chaotic and violent scenes of 19th-century New Orleans are overlaid with a dramatic, flashing, capitalized “YELLOW FEVER” text across the screen, as if to heighten the emphasis on uncontrolled panic.

Another aspect of declining social morality is linked to scapegoating; stigmatization and blame have long been connected with epidemics. It is well known that the Black Death brought mass persecution of Jews in various parts of Europe (*13*), and families were gathered up and burned alive. More recently, Muslims were blamed for poisoning water systems during the 1994 plague outbreak in Surat, India (*14*), and the HIV/AIDS pandemic from the 1980s led to extreme prejudice against homosexuals and intravenous drug users (*15*). Globalization and the fear that exotic diseases can be transported into modern urban environments (*16*), together with increased access to air travel (*17*), has to some extent heightened these kinds of concerns about scapegoating. Viruses know no borders and thus become easily entangled with contemporary anxieties over migration and refugees (*18*). Asian populations in Chinatowns of various Western cities were victimized in the wake of severe acute respiratory syndrome (SARS) (*19*), and studies performed in Hong Kong revealed that the public actually anticipated this kind of outcome (*20*).

Not surprisingly, then, popular culture has often tended to present epidemics as a foreign invasion (*2*,*21*), especially given the moral associations often drawn between disease and social and cultural phenomena through metaphors such as corruption, decay, and pollution (*11*). For example, in the Sherlock Holmes story *The Giant Rat of Sumatra* (not penned by Conan Doyle), Professor Moriarty prepares to import plague to Britain by acquiring infected rats from Southeast Asia (*5*). Accordingly, in some recent Hollywood films, the direction of disease movement is typically east to west, or at least from developing to developed countries, and plays on common stereotypes, including concepts of orientalization and othering (*4*,*22*). The film *Contagion* (2011, Steven Soderbergh), although widely lauded for its accurate depiction of the mechanisms of disease transmission and epidemiology, can be seen to neatly fit within Wald’s outbreak narrative framework, in which a pathogen is brought into the developed world after contact with migrants and visitors from lesser developed areas (*4*,*6*). Evie Kendal noted that the virus that hit the United States in *Outbreak* (1995, Wolfgang Petersen) was thought to have originated in Africa, whereas in *Contagion* and *The Crazies* (1973, George Romero), the disease originates in Asia (*4*).

In more recent years, however, the othering narrative has also taken new forms, in which an undeveloped or traditional society under threat of an epidemic is heroically saved by outside forces. This narrative can be seen in the popular film set in China, *Wolf Warrior II* [Zhan lang II] (2017, Jing Wu), which tries to favorably present Chinese attempts to control a fictional disease (lamanla) based on Ebola in Africa. In *The Painted Veil* (2006, John Curran), a 1920s rural village in China is unable to deal with a cholera outbreak until a British bacteriologist comes along to investigate and in the end selflessly gives his own life.

## Resistance and Normalcy during Epidemics Depicted in Cinema

Although epidemics can produce panicked responses that lead to the unraveling of the social fabric, this response is not the only outcome, as the literature (both historical and contemporary sociological) has argued (*13*,*23*). In a related way, some films have also shown that extreme reactions during epidemics often have a specific purpose, and, paradoxically, elements of unrest (e.g., resistance to authorities and violence) can be society’s way of attempting to return to a state of normalcy and cohesiveness. Indeed, although according to Michel Foucault, sickness and disease became an arena through which authorities and elites tried to exert social controls (*24*), epidemics can also become a context in which those lower down the social hierarchy vent frustrations, leading to conflict, often as a way of protecting freedoms or traditions and customary practices. Good early cinematic examples of lower class involvement include *The Citadel* (1937, King Vidor), with its scenes of working class miners resisting medical authority during a tuberculosis outbreak, and *1918* (1985, Ken Harrison), in which residents of a small Texas community resolutely try to continue their normal lives, functions, and networks despite the scale of deaths from influenza.

In recent times, more nuanced views about how societies respond to epidemics can be found in films, especially showing how extreme reactions tend to not be intrinsically associated with the disease itself but rather to be a way of dealing with top-down repression impinging on freedoms and liberties. Although perpetuating some elements of othering (*6*), the US-based film *Contagion* is a good example of a film that deals with social movements from below, in the process criticizing contemporary trends toward avarice and self-interest, which are further exposed during the epidemic. Throughout the movie, Soderbergh presents collective patterns of behavior during the epidemic, which lead to social discord. However, much of this unrest is connected to unsatisfactory responses to the disease by authorities. Most fears stem from an absence of information from reputable sources, leaving a vacuum for dubious intelligence to emerge on alternative platforms, where intrigue and speculation become almost comparable types of contagion. Overall, a tension emerges between the medical authorities’ advised procedure of forcible isolation and quarantine, which conflicts with very contemporary demands for maintenance of ordinary patterns of social networking and communication. This response has strong parallels in history, where communities have been shown to cling strongly to their sociability. Religious rituals such as the Janāza blessing continued unabated in the Middle East during Second Pandemic plagues (*25*), and the influenza pandemic of 1918–1920 has been cited as the foremost example in modern history of continued social bonds in times of excessive deaths (*26*).

Another film demonstrating similar issues is *Blindness* (2008, Fernando Meirelles), which deals with a fictional disease that causes epidemic blindness, leading to collective hysteria. Overall, the film considers the human capacity for prejudice, indifference, selfishness, and an easy resort to aggression and violence. As more citizens contract the disease, the normal functioning of society is upturned: strict government quarantines of the infected are imposed by use of physical force. Just as with Contagion, however, this action provokes unrest not connected to the disease but to perceived arbitrary and ruthless actions of elites and authorities. We see scenes in which resources such as food begin to be distributed inequitably, and people begin to exploit their positions by withholding food in exchange for other resources, including coerced sex. Most of the violent confrontation stems from hostilities between those subject to quarantines and those managing the confined environments.

These aspects of extreme disdain for the withholding of perceived societal freedoms are not found solely in Western films but in films from Asia too, such as the recent popular movie *Flu* (2013, Kim Sung-su), set in South Korea, which plays on recent experiences with SARS and influenza by focusing on responses to an outbreak of a fictional disease with parallels to the strain of avian influenza A(H5N1) virus. On the one hand, many scenes capture the frenzied breakdown of social norms. As first details of the disease emerge, the film’s protagonist, Ji-goo, is seen in a mall among people frantically making phone calls and rushing to leave the city. After the news is made public on national television, raising the category of the crisis to critical, citizens begin fighting for resources and even exploiting the chaos to loot a supermarket. On the other hand, however, we also see much more focused forms of unrest directed at certain social groups, especially in the process of implementing quarantines. Of note, when infected citizens are forcibly moved to camps, elements of compassion and cohesion develop among camp residents; the most violent reactions come from and are directed toward authorities managing the site. The film also reflects on distrust of elites and authorities with medical knowledge and power; rumors are spread that the infected are killed rather than treated. This same distrust is a theme seen in other films from China, such as *Shen yi bian que* (1985, Yin Cui) and *Fall of Ming* [Da Ming jie] (2013, Jing Wang), about real-life physicians Bian Que and Wu Youke, respectively. This theme again has broad parallels to history and the riots that occurred during 19th- and 20th-century cholera outbreaks, opposing medical staff and governments (*27*). Note that several films set in China and Hong Kong produced in the immediate aftermath of the 2003 SARS outbreak aimed to present an optimal governmental response: *Profoundly Affecting* [*Jingxing dingpo*] (2003, Jia Wang/Dong Shen), *Feidian rensheng* (2003, Wai-Man Cheng), and *Golden Chicken 2* [*Gam gai 2*] (2003, Leung Chun Chiu).

Aside from freedoms and liberties, other films have highlighted how societies sometimes move toward unrest—through either resistance to authorities or physical violence—as a response to perceived infringements on customary and traditional practices that have occurred during an epidemic outbreak. One of the clearest demonstrations of this response appears in the 2006 readaptation of *The Painted Veil*, which follows a British bacteriologist working to prevent the spread of cholera in a small village in rural China in the 1920s, set within a broader context of distrust through heightened nationalist tensions. In this film we see no signs of fear or panic from the local population, despite many deaths within the community; the inhabitants are more concerned about maintaining ordinary patterns of living and sociability. The villagers become incensed for the first time when the foreign doctor tries to move bodies from the cemetery to avoid infecting the water; the villagers believe that the deceased must be nearer to the river to move swiftly to the afterlife. The decision to remove all corpses and bury them immediately raises further fury because the villagers expect a certain period to elapse while the bodies are laid out in their homes before burial. Although we must be aware of the potential effects of the orientalist imagination when considering a film such as *The Painted Veil*, medical history points to its accuracy. In rural China during the 1920s and 1930s, elite medical reformers’ disregard for village internal politics and power dynamics limited the effectiveness of their public health prevention policies, as locals continued the same routines (*28*). The same issues also appear in older productions set in China; in *Horse Thief* [*Dao ma zei*] (1986, Tian Zhuangzhuang), set during a disease outbreak in Tibet in the 1920s, people living out in the steppe or grasslands continue their very same daily functions, stopping only to pray for the disease to cease.

## Conclusions

Cinema is not reality, and elements of fear leading to panic and chaos are likely to persist in dramatic representations of disease, purely for entertainment value. These representations may be problematic in 2 interrelated ways. First, “the image of a panicked mob makes exciting footage in disaster movies, but it obscures a broad range of possible public reactions” (*29*). Second, the public perception of how epidemic diseases behave is substantially influenced by the media and popular culture (*2*,*4*,*6*,*30*); parts of this perception are sometimes adopted as scientific facts (*31*,*32*). Similar issues have recently been brought to our attention regarding the widespread and incorrect diagnostic use of images of persons with a disease that is wrongly assumed to be plague (*33*,*34*) and media perpetuation of several recent misguided anxieties over how the Ebola virus is spread (*35*,*36*).

In this article, however, we have shown that many films focusing on social responses to epidemic disease outbreaks also shine a light on another side of how people react. Although on the one hand, fear and panic can be connected to a perceived breakdown in social morality, on the other hand, disruptive reactions can also work toward societal cohesion to protect freedoms, privileges, and customs under threat. By shifting between the macro scale of humanity or society to the micro scale of individual protagonists and relatable characters—including different social and demographic groups—films can, at times, do an excellent job of showing how disease responses are also shaped by hierarchical relationships (not just the demonstration of power from above but also the reception of this power from below). In the films mentioned in this article, a recurring lesson is the failure of imposed mass isolation techniques, largely resulting from weak compliance (*4*). These films also show situations similar to ongoing problems with Ebola in Africa today, where communal suspicions and distrust of the decisions of outside authorities are rife (*37*–*39*), violent resistance and outcry occur within communities (*40*), and localized attempts continue to maintain customary practices in terms of the treatment of the dead (*41*).
